# Are the body shape index, the body roundness index and waist‐to‐hip ratio better than BMI to predict recurrent pregnancy loss?

**DOI:** 10.1002/rmb2.12388

**Published:** 2021-05-21

**Authors:** Tunay Kiremitli, Sevil Kiremitli, Pasa Ulug, Kemal Dinc, Kemine Uzel, Yusuf Kemal Arslan

**Affiliations:** ^1^ Medical Faculty, Obstetrics and Gynaecology Department Erzincan Binali Yildirim University Erzincan Turkey; ^2^ Biostatistics Department Medical Faculty Erzincan Binali Yildirim University Erzincan Turkey

**Keywords:** a body shape index, body mass index, body roundness index, recurrent pregnancy loss, waist‐to‐hip ratio

## Abstract

**Purpose:**

Etiology could not be determined in approximately 50% of recurrent pregnancy loss cases, and it was named unexpected recurrent pregnancy loss(URPL). A body shape index(ABSI), body roundness index(BRI), and waist‐to‐hip ratio(WtHR) are new indexes that are superior to BMI in showing body fat distribution. We aimed to investigate the potency of ABSI, BRI, and WtHR in URPL, their superiority to BMI, and their suitability for clinical use.

**Methods:**

One hundred and thirty‐eight patients between the ages of 20‐40 who applied to our hospital for URPL between January 2016 and December 31, 2020 were included in our study. Weight, height, waist circumference, and hip circumference were measured, and indexes were calculated. Differences between the URPL and control groups were calculated using the IBM SPSS program.

**Results:**

There was a significant difference between the two groups for BRI, ABSI, and WtHR values, while there was no significant difference in BMI. BRI(4.4 ± 1.7vs3.9 ± 1.5), ABSI(0.08 ± 0.005 vs 0.078 ± 0.004), and WtHR(0.84 ± 0.06vs0.82 ± 0.05) values were higher in the URPL group. ROC analysis showed us that BRI, ABSI, and WtHR have a diagnostic value for URPL(*P* < .05). When indexes were above the cutoff values, RPL risk increased 3.59 times in ABSI, 2.26 times in BRI, and 2.9 times in WtHR(*P* < .05).

**Conclusions:**

The relationship between obesity and URPL can be explained more clearly by using effective indexes that show body fat distribution rather than BMI. Ethics committee approval was obtained from Erzincan Binali Yildirim University in 14.01.2021. Clinical Research Ethics Committee no: 01/01.

## Graphical abstract

A body shape index(ABSI), body roundness index(BRI) and waist‐to‐hip ratio(WtHR) are new indexes are superior to BMI in showing body fat distribution. ROC analysis show us that BRI, ABSI, WtHR have a diagnostic value for unexpected recurrent pregnancy loss (P < .05). The relationship between obesity and URPL can be explained more clearly by using ABSI, BRI and WtHR which showing body fat distribution rather than BMI.

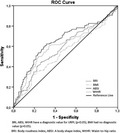

## 1 INTRODUCTION

The spontaneous termination of pregnancy before the 20th week and when the fetal weight is below 500 g is called “abortion,” and about 15%‐25% of known pregnancies will end in a miscarriage.^1^, ^2^ Recurrent pregnancy loss (RPL) has been defined as the loss of three or more consecutive pregnancies before the 20th gestational week.^3^ The rate of RPL ranges from 1‐5% of women of reproductive age.^4^ The etiology of RPL includes immunological, genetic, endocrine, anatomical, environmental factors, and infections. Despite all these investigations, etiology could not be determined in approximately 50% of RPL cases, and it was named unexpected recurrent pregnancy loss (URPL).^5^

Obesity is a chronic disease characterized by an increase in body fat mass since the energy taken into the body is greater than the energy consumed.^6^ Obesity is determined by the measurement of body mass index (BMI). The World Health Organization defined a BMI of 25 and above as overweight and 30 and above as obesity.^7^ In the literature, studies have reported that infertility, abortion, recurrent pregnancy losses, and failure rates in assisted reproductive techniques increase in obese patients.^8^, ^9^ It has been found that many chronic diseases, especially diabetes and cardiac diseases, are related to body fat distribution and fat percentage rather than the weight‐height ratio.^10^, ^11^ MRI, bio‐impedance analysis, air displacement plethysmography, and dual‐energy X‐ray absorptiometry are direct methods used to show body fat distribution. However, researchers have been canalized to new indirect methods that can be applied more easily, since direct methods are not practical in the clinic, their cost is high, and experienced personnel are needed for shooting and interpretation.^12^

It has been reported that waist circumference (WC) and waist‐to‐hip ratio (WtHR) are superior to BMI in showing cardiometabolic diseases.^13^ Based on this, an index named a body shape index (ABSI) was developed by Krakauer NY and Krakauer JC in 2012, and it was determined that it is superior to BMI and WC alone in showing premature mortality.^14^ The Body Roundness Index (BRI), which was developed in 2013 by Thomas et al. has modeled the human body as an ellipse and considered it in two axes: the major axis consisting of height and the minor axis consisting of waist and hip. They defined the degree of body roundness as "eccentricity" between 1 and 16. Values approaching 1 indicate thin and narrow elliptical‐shaped bodies, while values close to 16 indicate bodies with round and wide elliptical shapes.^15^ It has been reported that BRI is more sensitive than BMI and WC in predicting metabolic syndrome and dyslipidemia.^16^, ^17^

Ovarian reserve expresses the reproductive potential of the woman in terms of number and quality.^18^ Follicle stimulating hormone (FSH), estradiol (E_2_), antimullerian hormone (AMH) are some tests used for determining ovarian reserve.^19^ Obesity has been associated with a decreased ovarian reserve and impaired oocyte quality by affecting follicle functions and development.^20^, ^21^ Studies concluding that decreased ovarian reserve may cause RPL and infertility as a result of the effect on oocyte quality and number are available in the literature.^22^, ^23^

One of the important underlying factors of central obesity is leptin/adiponectin imbalance, which has been reported to be effective in determining the prognosis of diseases associated with abdominal obesity.^24^ Besides its role in fetal growth and development, leptin also has a modulator role for syncytiotrophoblates. It takes part in autocrine / paracrine events in implantation and the continuation of pregnancy.^25^ It was thought that the impairment of leptin balance and the resulting leptin resistance may be associated with poor reproductive performance and miscarriage.^26^ In light of this information, in this study, we aimed to investigate the potency of ABSI, BRI, and WtHR in URPL cases, as well as their superiority to BMI, their suitability for clinical use, and their effects on ovarian reserve.

## 2 MATERIALS AND METHODS

One hundred and thirty‐eight patients between the ages of 20‐40 who applied to our hospital for URPL between January 2016 and December 2020 were included in our study. Their files were reviewed retrospectively. The study was conducted in accordance with the principles of the Declaration of Helsinki. Ethics committee approval was obtained from Erzincan Binali Yildirim University in 14.01.2021. Clinical Research Ethics Committee no: 01/01.

Patients included had a normal hereditary, acquired thrombophilia panels and autoantibody tests, no abnormal maternal and paternal karyotyping, and had the normal results (after 12 hours of fasting) of glucose, TSH, prolactin, and vitamin D tests. All patients were tested for possible intrauterine pathology (endometrial polyp, submucous myoma, intracavitary septum) by transvaginal ultrasonography. Hysteroscopy was performed on suspected patients, and patients with pathology were excluded from the study. Patients with previous ovarian or uterine surgery, endometrioma, menstrual irregularity, polycystic ovaries, smoking or alcohol consumption, history of chemotherapy or radiotherapy, or genetic abnormalities were excluded from the study. The control group included 139 healthy, 20‐ to 40‐year‐old patients who had no previous abortion history, did not need assisted reproductive techniques to conceive, and had at least one live birth after 37 weeks of gestation.

### 2.1 Calculation of Anthropometric Indexes

The weight of the patients was measured in kg and height in cm. While the patients wore thin clothes for weight measurement, shoes were removed for height measurement. The weight was measured at approximately 0.1 kg. The height was measured at approximately 0.1 cm. Waist circumference was measured over bare skin, midway between the lower rib margin and the iliac crest at the end of expiration. Hip circumference was measured as the maximum circumference over the buttocks to the nearest 0.1 cm using a soft tape measure.

Women were classified into five BMI groups: underweight (<18.5 kg/m^2^), normal weight (18.5‐24.99 kg/m^2^), overweight (25‐29.99 kg/m^2^) obese (30‐39.9 kg / m2), and massive obesity (≥40kg/m^2^) in accordance with the WHO classification of BMI.^27^

Calculation formulas of BMI, WtHR, ABSI and BRI are;

$$\text{BMI}=\frac{\text{weight}(\text{kg})}{{\text{height}}^{2}\left(m\right)},$$

$$\text{WtHR}=\frac{\text{waist}\;\text{circumference}(\text{cm})}{\text{hip}\;\text{circumference}(\text{cm})},$$

$$\text{ABSI}=\frac{\text{wc}(\text{cm})}{\text{BMI}\frac{2}{3}\times \text{height}\frac{1}{2}},$$

$$\text{BRI}=364,2‐(365,5\times \sqrt{1‐\frac{{\left(\text{waist}\;\text{circumference}(cm)\right)}^{2}}{{\left(0.5\times \text{height}\right)}^{2}}).}$$

### 2.2 Statistical analyses

IBM SPSS version 21 (IBM Corp) was used for analyzing data. Descriptive statistics of continuous variables were presented as mean ± standard deviation, median (minimum‐maximum) value, and categorical variables as number (%). The compliance of the data to normal distribution was tested with the Shapiro‐Wilk test. The Mann‐Whitney *U* test was used when comparing continuous variables in groups. The Chi‐square test was used in the analysis of categorical variables. While testing the diagnostic value of the indices, ROC analysis was used, and area under curve (AUC) was presented with 95% confidence intervals (CI). Youden's index was used while determining the optimum cutoff value, and diagnostic accuracy criteria for the cutoff were presented. RPL risk was given as odds ratio (OR) according to the index cutoff points determined. A *P* value of <0.05 was significant in all statistical tests. While determining post hoc powers for primary outcomes (BRI, ABSI, WtHR), effect sizes were taken as 0.311, 0.442, and 0.362, respectively. Type‐I error was taken as 0.05 and post hoc powers found as 74.0%, 95.5%, and 85.1%, respectively. While determining the difference between groups, the sample size was adequate. For post hoc power calculation, G*power 3.1.9.2 was used.

## 3 RESULTS

The demographic and characteristics of both groups are shown in Table 1. The BMI had no statistically significant difference between groups (*P* = .276). According to BMI classification in the URPL group, 5 (3.6%) patients were underweight, 68 (49.3%) patients were normal weight, 39 (28.3%) patients were overweight, 23 (16.7%) patients were obese, and 3 (2.2%) patients were in the massive obese group. In the control group, 4 (2.9%) patients were underweight, 76 (54.7%) patients were normal weight, 37 (26.6%) patients were overweight, 20 (14.4%) patients were obese, and 2 (1.4%) patients were in the massive obese group. There was no significant difference in terms of BMI calcification in the subgroups (*P* > .05).

**TABLE 1:** The demographic and characteristics of URPL and control groups

	URPL Group (n: 138)	Control Group (n: 139)	*P*
Age	30.7 ± 4.93	29.7 ± 4.1	.106*
Gravida	4.0 (3‐8)	2 (1‐6)	<.001**
Parity	0 (0‐4)	2 (1‐6)	<.001**
Height (cm)	164 (145‐180)	163 (147‐180)	.319**
Weight (kg)	66 (44‐109)	67 (47‐120)	.087**
Waist circumference (cm)	88 (63‐124)	83 (63‐118)	.002**
Hip circumference (cm)	108.5 (83‐130)	103 (85‐129)	.039**
BMI	25.8 ± 5.1	25.0 ± 4.5	.276*
BRI	4.4 ± 1.7	3.9 ± 1.5	.016*
ABSI	0.08 ± 0.005	0.078 ± 0.004	<.001*
WtHR	0.84 ± 0.06	0.82 ± 0.05	.003*
AMH (ng/mL)	3.3 (0‐7.3)	4.6 (0.3‐8.2)	<.001**
AMH≤1	25 (18.1%)	18 (12.9%)	.235***
AMH>1	113 (81.9%)	121 (87.7%)	
FSH (U/L)	7.2 (3.8‐18.4)	7.1 (1.9‐24)	.132**
FSH<11	19 (13.8%)	15 (10.8%)	.45***
FSH≥11	119 (86.2%)	124 (89.2%)	
E_2_ (nmol/L)	46 (32‐82)	47 (29‐78)	.135**
E_2_<60	12 (8.7%)	13 (9.4%)	.849***
E_2_≥60	126 (91.3%)	126 (90.6%)	

Abbreviations: BRI, Body roundness index; ABSI, A body shape index; WtHR, Waist‐to‐hip ratio; URPL, Unexpected recurrent pregnancy loss.

* Independent samples *t* test was performed

** Mann‐Whitney *U* test was performed

*** Chi‐square test was performed.

54 (39.1%) patients in the URPL group had at least one live birth in their medical history, and 74% of these patients had a normal vaginal delivery. All patients in the control group had at least one live birth, and 71.9% of the patients had delivered vaginally. There was no statistically significant difference in terms of delivery type (*P* = .695).

When AMH≤1 and AMH>1 were compared, the number of patients with AMH≤1 was more common in the URPL group; the difference was not statistically significant (*P* = .235). Similarly, among the poor ovarian reserve markers, serum FSH≥11 and serum E_2_≥60 values did not show a significant difference between the URPL and control groups (*P* >.05) (Table 1).

As BMI increased, the number of patients with AMH≤1 ng/mL increased for both the URPL and control groups (*P* < .01). While AMH≤1 was observed in 8.3% of the patients with normal BMI, this rate increased to 25% in patients with BMI≥25 (*P* < .05). There was no significant difference between the BMI groups in terms of FSH≥11 and E_2_≥60 (*P* > .05).

As shown in Table 1, there was a significant difference between the two groups for the BRI, ABSI, and WtHR values, while there was no significant difference in BMI. BRI, ABSI, and WtHR values were higher in the URPL group.

**FIGURE 1:**
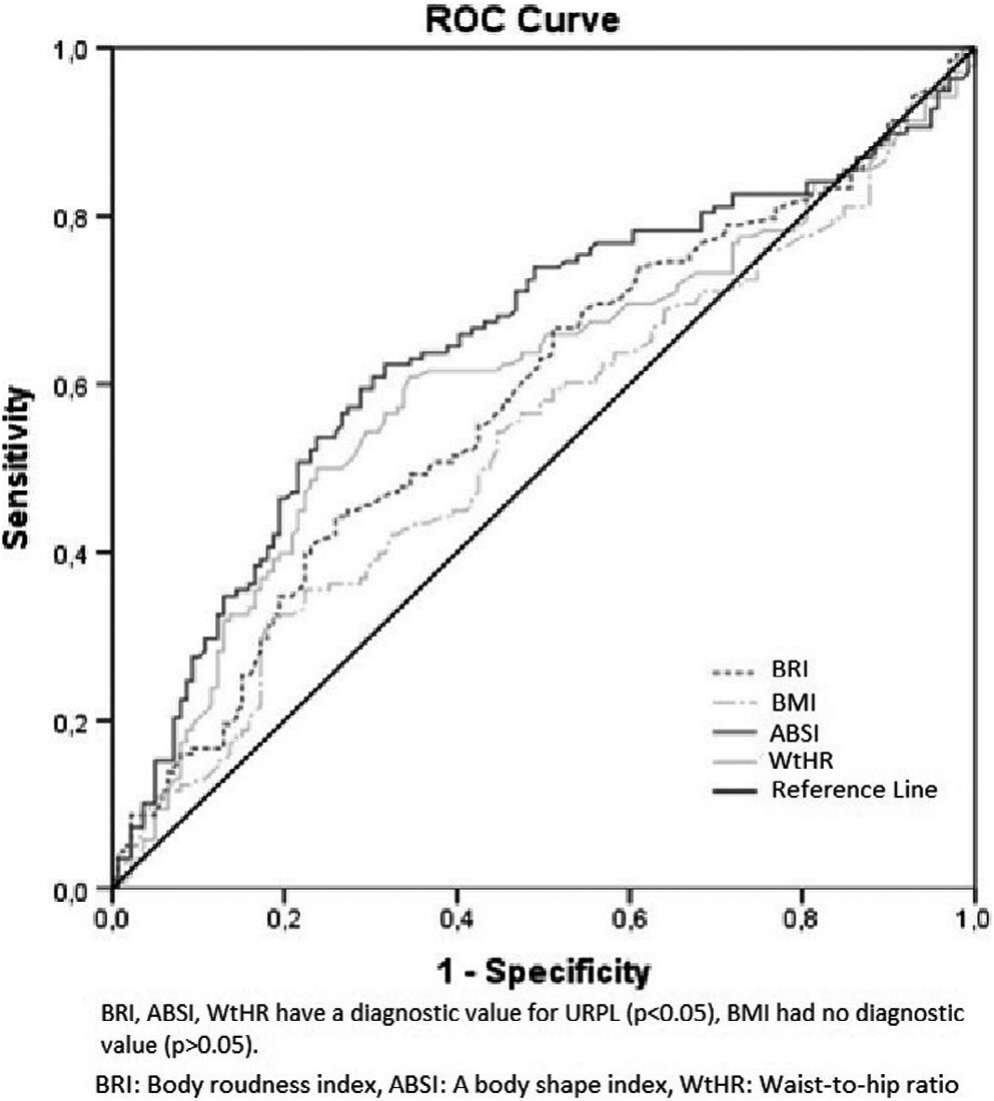
ROC curve of BRI, ABSI, WtHR and BMI: BRI, ABSI, WtHR have a diagnostic value for URPL (*P* < .05), BMI had no diagnostic value (*P* > .05)

ABSI, BRI, and WtHR were evaluated with ROC analysis; cutoff levels were determined, and AUCs were calculated. While it is seen as a result of ROC analysis that BRI, ABSI, and WtHR have a diagnostic value for URPL (*P* < .05), BMI had no diagnostic value (*P* > .05) (Figure 1)(Table 2).

**TABLE 2:** Diagnostic accuracy measures of indexes and ROC analysis results

	Cutoff	Sensitivity	Specificity	AUC (95%CI)	*P*
BRI	4.37	44.2%	74.1%	0.584 (0.517‐0.651)	.016
ABSI	0.079135	60.9%	69.8%	0.649 (0.583‐0.715)	<.001
WtHR	0.8178	60.9%	65.5%	0.605 (0.537‐0.672)	.003

Abbreviations: ABSI, A body shape index; AUC, Area under the curve; BRI, Body roundness index; CI, Confidence interval; WtHR, Waist‐to‐hip ratio.

It was observed that the BRI above the cutoff value of 4.37 increased the URPL risk 2.26 times (OR: 2.267, 95% CI: 1.365‐3.763, *P* = .002).

The cutoff value of ABSI was calculated as 0.079, and above this value, URPL risk increased 3.59 times (OR: 3.59, 95% CI: 2.184‐5.91, *P* < .001).

WtHR's cutoff value was 0.81, and URPL risk was calculated as a 2.9‐fold increase in patients with WtHR above the cutoff (OR: 2.949, 95% CI: 1.80‐4.80, *P* < .001).

In regions above the cutoff value for BRI, ABSI, and WtHR, the number of patients with AMH≤1 µg/L was higher and statistically significant (*P* < .05). However, an FSH level ≥11 U/L, a serum E_2_ level≥60 nmol/L did not have statistical significance in patients who were below or above the cutoff value (*P* > .05).

In normal BMI patients, the relations of the URPL and control groups with the BRI, ABSI, and WtHR cutoff values are shown in Table 3. The URPL rate had been significantly increased in the patients above the cutoff value for all three indexes (*P* < .05).

**TABLE 3:** The relationship of BRI, ABSI and WtHR with URPL in those with normal BMI

		URPL	Control	Total	*P*
BRI	Under zone of the cutoff	59	76	135	.01
	Upper zone of the cutoff	9	0	9	
ABSI	Under zone of the cutoff	26	56	82	<.01
	Upper zone of the cutoff	42	20	62	
WtHR	Under zone of the cutoff	29	61	90	<.01
	Upper zone of the cutoff	39	15	54	

Abbreviations: ABSI, A body shape index; BRI, Body roundness index; URPL, Unexpected recurrent pregnancy loss; WtHR, Waist‐to‐hip ratio.

## 4 DISCUSSION

This study demonstrates that ABSI, BRI, and WtHR are effective in predicting URPL, and demonstrates that indexes showing body fat distribution should be used more effectively in the etiology of URPL instead of or with BMI.

BMI, which is used for the diagnosis and classification of obesity, is insufficient for showing fat‐muscle separation and body fat distribution.^15^, ^28^Using different indexes instead of BMI in determining the risk of obesity‐related diseases has been the subject of multiple studies. The role of this new indexes in predicting coronary artery disease, diabetes mellitus, and metabolic syndrome has been investigated, and they have been found to be in a stronger relationship than BMI.^29^, ^30^, ^31^

RPL, which in 50% of cases is of unknown etiology, making it impossible for patients and physicians to determine future pregnancy outcomes, may cause serious emotional stress and depression for patients.^32^It is known that obesity increases the risk of first trimester and recurrent pregnancy loss. Therefore, studies have been conducted on the etiology of RPL, and its relationship with obesity has been examined using BMI.^33^

In this study, the relationship between BRI, ABSI, and WtHR with URPL, their potential superiority to BMI, and its relationship with ovarian reserve have been investigated. The relationship with URPL and an index (other than BMI) that may be effective in showing the diagnosis of obesity and fat distribution has not been investigated before in the literature. Our work is a first in this respect.

Obesity is thought to have a negative effect on female fertility by affecting the hypothalamic‐pituitary‐gonadal‐hormonal axis, oocyte quality, embryo development, and endometrial receptivity.^34^

According to Cavalcante et al. when the patients were classified according to BMI in the meta‐analysis, the relationship between the obese group and RPL was seen, but the risk was not determined in the overweight and underweight groups.^35^ Metwally et al. reported that recurrent pregnancy loss is more common in the obese and underweight groups when RPL patients are classified according to BMI.^36^ Lo et al. in their study using BMI groups, found that RPL risk increased in the obese group, while RPL risk did not change in the overweight and underweight group.^37^It has been reported that central obesity is more effective than BMI in terms of maternal and fetal complications.^38^However, we did not find any study in the literature showing the relationship between central obesity and RPL. Also, considering the difference between BMI classes and RPL, we thought it would be more decisive to study the relationship between URPL and central obesity and anthropometric indices that show better muscle‐fat separation, considering that various combinations of height and weight can reach the same BMI.

Some studies also found that WC and WtHR are better markers than BMI in obesity‐related diseases.^39^, ^40^ This is thought to be because WC and WtHR are related to unhealthy weight distribution and are more effective than BMI in showing central fat distribution.^41^ABSI, which was developed by using BMI and WtHR together, was found to be more effective in predicting the risk of premature death than BMI.^20^ Bawadi et al. stated in their study that ABSI has a higher predictive potency than BMI for diabetes mellitus.^42^

In metabolic syndrome, the potency of ABSI was found to be lower compared to BMI and WtHR, while BRI was reported to be the most effective index in predicting metabolic syndrome.^16^ In another study, BRI was found to have the highest capacity to define diabetes mellitus, while BMI was found to be the least associated.^30^In this study, where we investigated the relationship between URPL and indexes that were easily calculated in clinical practice, it is striking that WtHR, BRI, and ABSI values were significantly higher and diagnostic power in the URPL group, while there was no difference between the groups in terms of BMI value and BMI classification. We found ABSI has the best predictive ability for URPL, and if ABSI was above the cutoff value, URPL risk increased 3.59 times. WtHR above the cutoff value increased the risk 2.9 times, and BRI above the cutoff value increased the URPL risk 2.26 times.

In the normal BMI class, 67.7% of the patients above the cutoff value calculated for ABSI and 72.2% of the patients above the cutoff value calculated for WtHR were in the URPL group. It was interesting that while nine patients were followed in patients with normal BMI above the BRI cutoff value, all of them were in the URPL group. In light of these results, the risk of URPL must increase with the accumulation of body fat distribution in the central region, even in patients who are calculated as a normal BMI group and who are thought to have excluded the effect of obesity on URPL, and we think that this should be considered.

It has been reported that diminished ovarian reserve (DOR) can be effective on RPL by affecting oocyte quality and oocyte number.^43^ In a meta‐analysis results, it was stated that there is a relationship between DOR and RPL, especially URPL. According to the results of the same study, it has been reported that low AMH levels (<1 ng/mL) and RLP are related, and the relationship between FSH and E2 values is not clear.^44^Atasever et al. found in their study that low AMH and high FSH values were associated with RPL.^23^ In another study, a significant relationship was found between RPL and AMH, but no significant relationship was found with FSH.^22^

The mechanism of action of obesity on ovarian reserve has not been clearly explained in the literature. The results differ according to the reserve tests used, patient population, and obesity classification. Moslehi et al. reported in their meta‐analysis that there is a negative relationship between obesity and AMH, but not with FSH.^21^ They concluded that the reduction of the ovarian reserve in obese patients with RPL history is in a closer relationship with AMH than the other reserve tests. In our study, AMH values were found to be significantly lower in the URPL group. While the number of patients with AMH≤1 ng/mL was higher in the URPL group, this difference was not statistically significant. FSH and E_2_ values did not differ significantly between the groups.

Although there was a negative relationship between AMH and all indexes in our study, no significant relationship was found for FSH and E_2_. When we look at the relationship between the AMH value and the indexes, lower AMH values were observed in the obese and massively obese patient group compared to the patients with a normal BMI, while the number of patients with AMH≤1 ng/mL was higher. The number of patients with AMH≤1 ng/mL showed a significant difference above and below the cutoff value calculated for BRI, ABSI, and WtHR. AMH decreased as the index values increased. As the bodyweight rose above normal, ovarian reserve and quality decreased; we think that the detected RPL increase depends on this. However, we did not detect that the central distribution of obesity has a greater effect than obesity alone on reserve tests. In other words, the power of other indices to affect reserve tests was not higher than BMI. In future studies with more patients, the effect of fat distribution on ovarian reserve in the URPL patient group using other ovarian reserve tests will be seen more clearly by determining age ranges and cutoff values.

The limited number of samples, especially for cutoff values calculated for indices, and relatively few patients in the underweight and massive obese groups as a result of BMI classification are the limitations of the study.

## 5 CONCLUSION

In conclusion, URPL is a devastating situation whose etiology has not fully resolved, and the subsequent pregnancy outcomes are difficult to predict by patients and clinicians. In this study, we demonstrated that BRI, ABSI, and WtHR have diagnostic value for URPL, as in obesity‐related diseases. We think that the relationship between obesity and URPL can be explained more clearly by using more effective indexes in showing body fat distribution rather than BMI. It should be kept in mind that central obesity is associated with an increased risk of pregnancy loss even in patients with normal BMI for URPL, whose etiology is questioned in every detail. Therefore, central region weight control and prevention of central obesity should be recommended to both patients with high BMI groups and patients with normal BMI groups.

## CONFLICT OF INTEREST

Tunay Kiremitli, Sevil Kiremitli, Pasa Ulug, Kemal Dinc, Kemine Uzel and Yusuf Kemal Arslan declare that they have no conflict of interest.

## AUTHOR CONTRIBUTIONS

Tunay Kiremitli, Sevil Kiremitli involved in conception and design. Kemal Dinc involved in acquisition of data. Yusuf Kemal Arslan involved in analysis and interpretation of data. Sevil Kiremitli, Kemine Uzel involved in drafting the article. Pasa Ulug involved in revising it critically for important intellectual content. Tunay Kiremitli involved in final approval of the version to be published. We declare that this work was done by the authors named in this article and all liabilities pertaining to claims relating to the content of this article will be borne by the authors. All authors read and approved the manuscript for publication.

## ETHICAL APPROVAL

All procedures followed were in accordance with the ethical standards of the responsible committee on human experimentation (institutional and national) and with the Helsinki Declaration of 1964 and its later amendments. Ethics committee approval was obtained from institutional review board of Erzincan Binali Yildirim University in 14.01.2021. Clinical Research Ethics Committee no: 01/01.
